# Editorial: From sex differences in neuroscience to a neuroscience of sex differences: new directions and perspectives

**DOI:** 10.3389/fnins.2015.00330

**Published:** 2015-09-23

**Authors:** Belinda Pletzer

**Affiliations:** ^1^Department of Psychology, Paris-Lodron-University SalzburgSalzburg, Austria; ^2^Centre for Cognitive Neuroscience, Paris-Lodron-University SalzburgSalzburg, Austria

**Keywords:** sex differences, sex hormones, menstrual cycle, hormonal contraception, menopause, neurotransmitters, stress hormones, sex role

While we are looking back at a century of behavioral research on sex differences in cognition and emotion, sex differences have for a long time been disregarded as a confound in neuroscience.

Several researchers still argue that sex differences in cognition are overall small and negligible (Hyde and Linn, 1988, 2006; Hyde, 2005, 2006). Indeed the data so far are by no means consistent. These inconsistencies are to a large part attributable to small sample sizes and low power on the one hand and a large variation in methodologies on the other hand. This is even more true for studies on menstrual cycle and hormonal contraceptive dependent effects on the brain, as will be pointed out in this Research Topic (Pletzer and Kerschbaum, 2014; Sundström Poromaa and Gingnell, 2014).

However, another reason that these inconsistencies still exist may also be that neuroscience, looking at the topic from the angle of political correctness, has refrained from studying sex differences in the brain in more detail. One major goal of this Research Topic is to change this view and look at the topic from a different angle:

Women don't have to be like men to be treated as equal. Women have a right to be women and if that includes being different from men, women have a right to be different from men and—importantly—also vice versa. After all, innovation stems from diversity (e.g., Hewlett et al., 2013). Furthermore, women have a right to understand these differences as well as the hormonal changes that not only their body, but also their brain, goes through during their lifetime. So it's important that neuroscience starts paying more attention!

The aim of this Research Topic is to point out directions and perspectives on how to resolve inconsistencies in sex difference research. The idea is to move from scattered findings on sex differences in the brain to a neuroscience of sex differences that will help researchers to understand and predict sex differences in their findings and integrate them into their theories.

From the beginning of sex difference research, it has been hypothesized that sex differences in behavior are at least in part driven by hormonal influences on the brain either during development (organizational) or later in life (activational) (e.g., Kelly et al., 1999). Of course, not all sex differences can be attributable to sex hormones, but genetic, epigenetic, and chromosomal effects also play an important role, as pointed out by Ivanka Savic' contribution to this topic (Savic, 2014). However, sex hormone influences have been hypothesized to explain that variation in performance is higher in women than in men (e.g., Hausmann and Güntürkün, 1999). Therefore, sex hormones are of special interest to this topic as one important factor that might explain inconsistencies between sex difference studies.

Accordingly, the idea for this Research Topic was born during a study on sex differences in number processing. In a number bisection task, we found for two behavioral effect that sex differences in brain activation patterns were present during one cycle phase (follicular or luteal), but not during the other (Pletzer et al., 2011). In a follow-up study using the same task we furthermore recognized that women using hormonal contraceptives differed from naturally cycling women in the same way as men (Pletzer et al., 2014). These findings have important methodological implications for research on sex differences.

First, I propose here, that sex differences should always be investigated by comparing men to naturally cycling women, i.e., naturally occurring sex differences. The question of hormonal contraceptive dependent influences on brain and behavior is related, but separate.

Given that in developed countries about half of all women below the age of 45 rely on some method of hormonal contraception (Guttmacher Institute, 2015), hormonal contraception may represent a major confound in the study of sex differences. Only a handful of researchers have so far investigated hormonal contraceptive dependent effects on cognition, brain structure or brain function and only few consistencies arise. Within this topic, these findings are reviewed, potential mechanisms of action of hormonal contraceptives on brain and behavior are discussed and perspectives for research on hormonal contraceptive dependent effects are suggested (Pletzer and Kerschbaum, 2014).

Second, I argue that differences between men and naturally cycling women cannot be investigated without taking menstrual cycle phase into account. Despite an early interest in menstrual cycle dependent influences on mood (Frank, 1931), menstrual cycle dependent changes in cognitive functions have only been investigated for about 25 years now, starting with a pioneer study by Hampson (1990a,b). Only few studies have however investigated menstrual cycle dependent changes using neuroimaging methods. In this topic, menstrual cycle dependent changes in brain and behavior were carefully reviewed by Sundström Poromaa and Gingnell (2014). However, here again, results on the direction of changes as well as on the particular hormones responsible for the changes are inconsistent and more detailed studies are lacking. Possible reasons include that menstrual cycle research is costly and time consuming. This is due to difficulties in the recruitment of women, who do not use hormonal contraceptives, on the one hand, and large drop-outs on the other hand due to inaccurate self-reports of menstrual cycle phase. In order to clearly differentiate between the effects of estradiol and progesterone on brain functions, it is of uttermost importance to test in clearly defined and rather narrow time windows of a woman's individual menstrual cycle and ideally test at three different time-points: menses, pre-ovulation, and mid-luteal. Only few researchers have so far realized such a design (e.g., Weis et al., 2008; De Bondt et al., 2015). Within this Research Topic, one contribution presents for the first time neuroimaging data of a naturally cycling woman accompanied over four menstrual cycles (Arélin et al., 2015).

Other exploratory approaches include a comparison of high (mid-luteal) and low (early follicular) phases in within- as well as between-subjects designs. Both approaches have been successfully realized within this Research Topic in order to explore menstrual cycle dependent effects on reward processing and decision making (Derntl et al., 2014; Reimers et al., 2014).

However, especially changes in brain function and behavior due to the pre-ovulatory estradiol peak have recently come to attention (Jacobs and D'Esposito, 2011). From an evolutionary perspective, such pre-ovulatory changes are of particular interest for sexual selection. Jacobs found, that the pre-ovulatory estradiol peak particularly influences dopamine-dependent cognitive functions. Behavioral and methodological implications of this estradiol-dopamine interaction have been outlined within this topic by Colzato and Hommel (2014). Examining the interaction between sex hormones and relevant neurotransmitter systems is one important step toward a neuroscience of sex differences. By combining the knowledge from cellular, molecular, and animal research on how sex hormones interact with neurotransmitter systems with the knowledge of which cognitive functions these neurotransmitters contribute to, we can formulate clear hypotheses on how sex hormones might influence a certain behavior. Therefore, this topic includes an extensive review on such sex hormone-neurotransmitter interactions (Barth et al., 2015). Such interactive approaches would also allow us to reduce the costly and complex menstrual cycle designs outlined above to those phases where the hypothesized interactions are to be expected.

The same argumentation can be applied to the interaction between sex hormones and other hormone systems. Sex and stress hormone interactions (Kirschbaum et al., 1992; Andreano et al., 2008) have especially drawn attention due to their potential role for the vulnerability to mental health disorders, such as depression. Two articles within this Research Topic focus on this problematic, focusing on both the organizational effects (Goldstein et al., 2014) and the activational effects (Gobinath et al., 2014) of sex hormones. Furthermore, the article of Sorwell and colleagues within this topic addresses sex hormone interactions with the anti-stress hormone DHEA (Sorwell et al., 2014).

Importantly, in both domains—sex hormone neurotransmitter interactions (Barth et al., 2015), and sex hormone—stress hormone interactions (Gobinath et al., 2014; Goldstein et al., 2014)—the articles are organized along hormonal transition periods across the female life-span. Everything outlined so far implicitly referred to sex differences between men and women in their reproductive years. However, periods of much more extreme hormonal changes than during a normal menstrual cycle, such as puberty, pregnancy, and menopause, may provide us with important insights on how sex hormones affect a woman's brain and behavior. Therefore, post-menopausal functional changes have been reviewed separately within this topic (Comasco et al., 2014).

Thus, we have extensively captured potential variations in brain function and behavior due to hormonal status in the female group focusing mainly on the sex hormones estradiol and progesterone. However, several sex differences have early on been attributed to the organizational and activational effects of testosterone, particularly in the male group. Therefore, when studying sex hormone modulation of sex differences, testosterone levels should also be taken into account. Within this topic, testosterone actions on the brain have been reviewed by Celec et al. (2015). Testosterone and its metabolites also receive particular attention in the articles contributed by Sorwell et al. (2014), as well as Krajnik et al. (2014).

Summing up, sex difference research should profit in the future methodologically from taking into account the hormonal status of participants, especially in the female group and theoretically from more straightforward research questions and clearly formulated hypotheses by taking into account for example the interactions between sex hormones and neurotransmitter systems as well as other hormone systems. Here, I also propose that one direction sex difference research should aim at in the future is more integrative approaches, explaining sex differences across a variety of behaviors. In one of my own articles I outline the idea that sex differences might stem from a common principle in brain organization, i.e., the lateralization of brain functions (Pletzer, 2014). Sex differences in hemispheric asymmetries have long attracted attention and are also in the focus of Savic's contribution to this Research Topic (Savic, 2014). My article thereby focuses less on particular abilities than on cognitive strategies in men and women (Pletzer, 2014).

Furthermore, in our latest contribution to the topic we try to pick up the theme introduced in the beginning of this Editorial. How strong are sex differences really and are they worth investigating? One argument against sex difference research has always been that the variation within men or women is much larger than the differences between men and women. As outlined in the previous paragraphs there are quite a number of ways how sex hormones, might contribute to this within-group variation. However, it might also be interesting for researchers to capture this variation within the male and female groups. We therefore dedicated our latest contribution to the topic of sex role orientation and the question how we can assess and which factors affect the individual maleness or femaleness of a person (Pletzer et al., 2015). Because, in the end, sex may be one important factor influencing our behavior, but not only men and women differ. Every individual is different and should be accepted as such.

## Conflict of interest statement

The author declares that the research was conducted in the absence of any commercial or financial relationships that could be construed as a potential conflict of interest.
